# A comprehensive review on the formation and mitigation of polycyclic aromatic hydrocarbons (PAH4) in edible oils: From oilseeds to oils

**DOI:** 10.1016/j.crfs.2025.101151

**Published:** 2025-07-21

**Authors:** Jiao-jiao Yin, Xiao-ming Jiang, Yi Nie, Wu Zhong, Xing-he Zhang, Pan Gao, Dong-ping He

**Affiliations:** a Key Laboratory of Edible Oil Quality and Safety for State Market Regulation, Wuhan Polytechnic University, 68 Xuefu South Road, Changqing Garden, Wuhan, 430023, China; b Key Laboratory for Deep Processing of Major Grain and Oil of Ministry of Education in China, College of Food Science and Engineering, Wuhan Polytechnic University, 68 Xuefu South Road, Changqing Garden, Wuhan, 430023, China; c Wuhan Institute for Food and Cosmetic Control, 1137 Jinshan Avenue, Wuhan, 430040, China

**Keywords:** Polycyclic aromatic hydrocarbon, Generation, Mitigation, Cooking, Oilseeds, Oils

## Abstract

Edible oils are susceptible to contamination by polycyclic aromatic hydrocarbons (PAHs), particularly PAH4 compounds, which include Benz [a]anthracene, Chrysene, Benzo [b]fluoranthene, and Benzo [a]pyrene, all of which are recognized for their toxic and carcinogenic properties. This review examines the mechanisms underlying the formation of PAH4 in edible oils, with a particular focus on the transformation process from oilseeds to oils. Factors influencing the formation of PAH4 include environmental contamination, the composition of fatty acids, and processing methods such as extraction, refining, and cooking. High-temperature techniques, including roasting, frying, grilling, and baking, facilitate PAH4 formation through lipid oxidation and thermal decomposition. Conversely, low-temperature and short-duration treatments, such as cold pressing, along with refining processes, effectively reduce PAH4 levels in oils. Although detection methods such as High-Performance Liquid Chromatography (HPLC) and Gas Chromatography-Mass Spectrometry (GC-MS) are reliable, they are also costly and time-consuming. In contrast, methods such as fluorescence spectroscopy, electrochemical sensors, and Surface-Enhanced Raman Scattering (SERS)-based optical sensors are more appropriate for on-site rapid detection. To reduce PAH4 concentrations, it is advisable to select raw materials with minimal contamination, utilize cold pressing or refining techniques during processing, and choose oils that are low in polyunsaturated fatty acids. Additionally, employing low-temperature cooking methods, such as steaming or boiling, is recommended. The incorporation of antioxidants during both processing and cooking can further mitigate PAH4 levels. This systematic review offers specific guidance for oils production and food safety monitoring, thereby enhancing the safety and quality of edible oils in the market.

## Highlights

• PAH4 formation in oils is closely related to fatty acid composition.
• High-temperature increases PAH4 levels via lipid oxidation and thermal decomposition.
• Refining processes effectively reduce PAH4 levels in oil.
• Antioxidants can effectively reduce PAH4 formation.

## Graphical abstract

**Figure:**
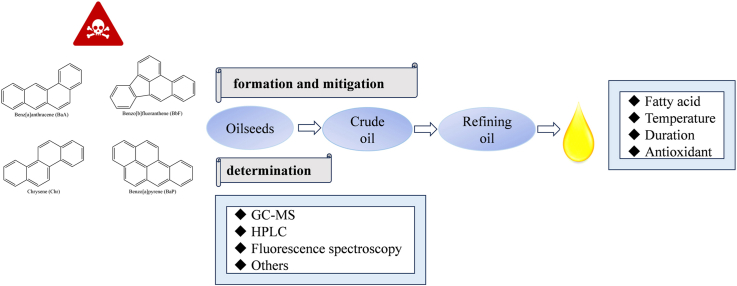

## 1 Introduction

**Fig. 1:**
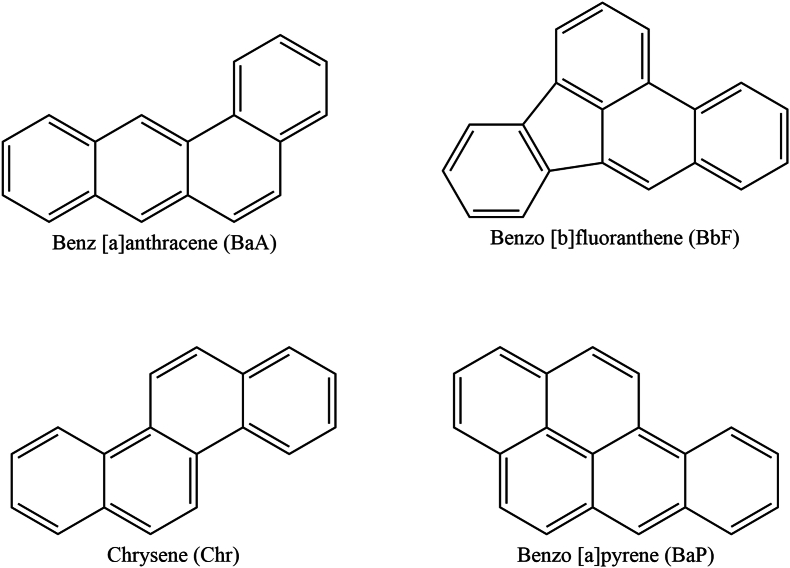
The structures of PAH4.

Polycyclic aromatic hydrocarbons (PAHs) are a class of toxic contaminants composed of two or more condensed aromatic rings, known for their high persistence and potential carcinogenicity, posing a serious threat to human health through food chain (Patel et al., 2020). The widespread presence of PAHs and their potential health risks have attracted considerable interest from global organizations like the European Food Safety Authority (EFSA) and the International Agency for Research on Cancer (IARC). With over 200 known PAHs, they can be categorized into heavy PAHs (H-PAHs, 5–6 rings) and light PAHs (L-PAHs, 2–4 rings) based on the number of rings. Among the various PAHs, Benzo [a]pyrene (BaP) is of particular concern due to its high toxicity. However, EFSA's 2008 assessment suggested that the PAH4, consisting of BaP, Benz [a]anthracene (BaA), Benzo [b]fluoranthene (BbF), and Chrysene (Chr), are more suitable as a limit indicator for PAHs in food compared to BaP alone (EFSA, 2008). The structures of PAH4 are illustrated in Fig. 1. In 2011, EU regulations stipulated that the BaP content in edible oils should not exceed 2 μg/kg, and the total PAH4 contents should not exceed 10 μg/kg (Commission of the European Communities, 2011). Besides, the German Society for Fat Science recommended limits of 5 μg/kg for H-PAHs and 25 μg/kg for total PAHs (Payanan et al., 2013). However, in China, the BaP content in edible oils should not exceed 10 μg/kg (GB 2762-2022, 2022).

It has been reported that for non-smoking and non-occupationally exposed individuals, 88 %–98 % of PAHs exposure originates from food (Wen et al., 2017). The complexity of the food system, with the presence of lipids, proteins, and carbohydrates, may contribute to the endogenous formation of PAH4 compounds (Zhang et al., 2021). Lipids, as the primary nutrients in food and the heating medium in food processing, play a crucial role in human health and food flavor. However, due to the lipophilic nature of PAH4, they tend to accumulate in high-fat foods and the generation of PAH4 during processing and cooking is closely associated with the presence of fats (Gao et al., 2024; Xu et al., 2024). Thus, approximately 50 % of the absorption and dietary intake of PAH4 from food is attributed to oils and fats contaminated with PAH4 (Wu et al., 2020).

The sources of PAH4 in fats mainly stem from two factors: exogenous contamination and endogenous processing generation. Exogenous contamination primarily refers to the exposure of raw materials to PAH4 pollution in the environment during growth, harvesting, drying, and storage stages. On the other hand, endogenous contamination is mainly concentrated in the food processing process, especially in high-temperature processing stages (such as baking, pressing, refining, frying, smoking, etc.), where incomplete combustion and thermal decomposition reactions lead to the generation of a large number of PAH4 (Ji et al., 2022; Lee et al., 2020).

In food, lipids are predominantly present as triglycerides. When subjected to thermal oxidation, triglycerides break down into diglycerides, monoglycerides, and free fatty acids. These lipid constituents undergo oxidation and decomposition concurrently during heat treatment, resulting in the fragmentation of lipid molecules into smaller molecular entities (Choe and Min, 2006). Studies have shown that lipid oxidation during food processing promotes the generation of PAH4 compounds. This process is particularly relevant in high-temperature cooking methods (such as frying, grilling, and baking), where lipid decomposition may result in the formation of harmful compounds. The relationship between lipid oxidation and PAH4 formation is complex, involving various chemical reactions that occur when lipids are heated. For instance, the degradation of lipid hydroperoxides can lead to the generation of conjugated carbon radicals, alcohols, aldehydes, and olefins, which serve as precursors for PAH4 through intramolecular cyclization and Diels-Alder reactions (Xu et al., 2024). Additionally, oils rich in polyunsaturated fatty acids (PUFAs) are particularly prone to oxidation, leading to increased levels of lipid oxidation products (LOPs) at high temperatures, thereby generating more PAH4 (Zhuang et al., 2022). Furthermore, the presence of unsaturated fatty acids in the lipid matrix can significantly increase the levels of cholesterol oxidation products (COPs) and PAHs during the heat processing (Min et al., 2015).

Current reviews focus on the distribution and contents of PAHs in edible oils (Ji et al., 2023; Sánchez-Arévalo et al., 2020), as well as the impact of different processing methods such as heating, drying, roasting, frying, and smoking on PAHs formation (Palade et al., 2023; Shen et al., 2023; Xu et al., 2023). However, there is currently a lack of systematic summarization on the formation mechanism of PAH4 in edible oils, especially from oilseeds to oils. This review focuses on the critical area of edible oils, providing an in-depth summary of the formation of PAH4 during the conversion process from oilseeds to oils. It comprehensively compares the differences in PAH4 formation under different processing methods and thoroughly summarizes the detection techniques and prevention strategies for PAH4. Through this systematic review, the aim is to provide more targeted guidance for the production and food safety supervision in the oil industry.

## 2 Generation of PAH4 in oils

The presence of PAH4 in edible oils primarily stems from both raw materials including oilseeds and processing conditions. Environmental pollution serves as a key factor contributing to PAH4 contamination in oilseeds, with sources including air, water, and soil pollution (Domingo and Nadal, 2015; Shi et al., 2016b). In addition to environmental factors, the processing of oils, particularly high-temperature treatments such as pressing and refining, can also lead to the formation and increase of PAH4.

### 2.1 Contents of PAH4 in different oilseeds and oils

The PAH4 contents in different oilseeds and edible oils are summarized in Table 1. Wen et al. (2017) analyzed the contents and distribution of PAH4 in 95 samples of 18 different oilseeds from various regions in China. The concentration of PAH4 in all samples ranged from 1.1 to 74.6 μg/kg. Furthermore, the ranges of PAH4 content in oilseeds of sesame, peanut, sunflower seed and walnut, which could be eaten directly, were 2.4–32.7, 5.4–52.0, 3.1–5.4, and 1.1–4.0 μg/kg, respectively (Wen et al., 2017). Additionally, Shi et al. (2016b) conducted a comparative assessment of the levels of BaP and PAH4 present in both oilseeds and the corresponding crude oils extracted from these seeds. The results revealed that the concentrations of BaP and PAH4 in the crude oils were 1.1 and 1.2 times higher, respectively, than those observed in the oilseeds. These outcomes suggest that oilseeds are susceptible to varying degrees of PAH4 contamination, with the PAH4 levels in oilseeds being transferred to the oils extracted from them.

**Table 1:** Mean contents of PAH4 (μg/kg) in different oilseeds and edible oils.

	Country	BaA	Chr	BbF	BaP	PAH4	Reference
Oilseeds							
Peanut	China	6.4	4.5	4.8	3.1	18.9	Wen et al. (2017)
Rice bran		1.9	1.3	1.8	1.4	6.3	
Rapeseed		5.7	6.3	7.2	4.8	24.0	
Soybean		1.5	1.7	1.8	0.9	6.0	
Sesame		3.2	3.3	3.3	2.3	12.1	
Sunflower seed		0.8	2.4	0.8	0.3	4.3	
Walnut		0.6	0.7	0.5	0.7	2.5	
Peanut with skin	China	1.45	1.08	1.13	1.16	4.82	Ji et al. (2020)
Peanut without skin		5.21	5.81	5.71	4.50	4.98	
**Oil type**							
Blended oil	Iran	0.34	0.08	0.54	0.29	1.24	Piravi-Vanak et al. (2024)
Coconut oil		1.1	1.24	1.73	0.31	4.40	
Frying oil		0.6	0.23	0.5	0.25	1.70	
Sunflower oil		0.2	0.2	0.76	0.18	1.33	
Sesame oil		0.8	0.30	0.30	0.30	1.78	
Olive oil		0.44	0.28	0.17	0.16	1.08	
Coconut oil	Brazil	1.75	2.34	1.29	1.22	6.60	da Silva et al. (2018)
Safflower oil		0.68	1.23	<1.00 (LOQ)	0.40	2.94	
Evening primrose oil		6.12	11.24	1.94	0.63	19.93	
Linseed oil		1.11	2.01	<1.00 (LOQ)	0.34	4.06	
Waste frying oil	China	0.85–2.74	1.58–5.25	1.36–7.83	1.09–6.60	6.10–18.62	Sun and Wu (2020)
Canola oil		1.99	3.84	2.03	2.82	11.18	
Sunflower oil		1.31	2.68	1.12	1.39	6.52	
Corn oil		3.06	6.04	2.70	3.03	14.19	
Rapeseed oil	China	1.40	1.73	2.62	1.49	7.24	Shi et al. (2016a)
Soybean oil		1.27	2.16	1.59	0.80	5.82	
Olive oil		0.49	1.31	0.63	0.46	2.88	
Peanut oil		4.09	5.09	4.37	2.40	15.95	
Sunflower oil		0.80	1.40	1.92	1.07	5.19	
Corn oil		1.34	2.62	2.02	0.97	6.95	
Sesame oil		1.56	2.86	2.02	1.04	7.49	
Cottonseed oil		2.50	3.34	4.58	2.42	12.84	
Rice bran oil		0.55	1.20	3.67	2.10	7.53	
Sunflower oil	Syrian	3.37	3.32	3.47	1.71	11.9	Krajian and Odeh (2018)
Soybean oil		2.55	2.11	2.08	1.59	8.35	
Corn oil		2.40	1.73	1.57	1.93	7.63	
Rice bran oil	South Korea	0.40	0.39	0.24	0.29	–	Choi et al. (2022)

Studies have indicated that the fatty acid composition of oilseeds plays a crucial role in the generation of PAH4. Analysis of the fatty acid composition in peanuts, sesame, sunflower, rapeseed, and flaxseed before and after roasting revealed a close correlation between the changes in fatty acid composition during roasting and the PAH4 contents in the extracted oils (Ji et al., 2022). Specifically, oilseed crops with higher levels of polyunsaturated fatty acids (PUFAs) are more prone to producing H-PAHs under high-temperature roasting conditions, while those with higher levels of monounsaturated fatty acids (MUFAs) and saturated fatty acids (SFAs) tend to generate L-PAHs. For instance, peanuts, sesame, and rapeseed exhibit significantly higher levels of L-PAHs compared to H-PAHs, attributed to their higher MUFA and SFA content, particularly their low PUFA/SFA ratio (P/S index). In contrast, sunflower and flaxseed, with higher PUFA contents (P/S index of 5.22 and 6.37, respectively), show relatively higher levels of H-PAHs (Ji et al., 2022). This is due to the thermal cracking of fatty acids in oilseeds under high-temperature roasting conditions, leading to the formation of hydroperoxides, which can undergo free radical recombination and Diels-Alder reactions to form relatively stable PAHs. Particularly, linoleic acid and linolenic acid in PUFAs are prone to polymerization reactions at high temperatures, forming benzene ring-containing monomers that serve as precursors to PAH4 (Chen and Chen, 2001; Xie et al., 2024). Kubátová et al. (2012) investigated the pyrolysis products of triglycerides present in vegetable oils when subjected to high-temperature conditions. Their findings revealed a significant presence of cyclized alkanes, with approximately 20 % of these compounds showing a strong correlation with the Diels-Alder mechanism. Furthermore, the study identified the involvement of intermolecular and intramolecular alkyl and alkenyl radicals in the formation of these cyclized alkanes (Kubátová et al., 2012).

As shown in Tables 1 and it could be seen that flavorful oils such as peanut oil and sesame oil exhibit higher PAH4 levels, which possibly due to direct high-temperature roasting and incomplete refining processes (Ji et al., 2022; Shi et al., 2016a). To preserve the flavor of peanut oil and sesame oil, only simple degumming processes are employed during refining, resulting in residual PAH4 in the oils. In contrast, soybean oil and sunflower seed oil show lower PAH4 contents after refining (Piravi-Vanak et al., 2024; Shi et al., 2016a). Additionally, although coconut oil is rich in saturated fatty acids, its PAH4 contents are often high (da Silva et al., 2018; Piravi-Vanak et al., 2024), attributed to the potential generation of PAH4 from its raw materials during the drying process.

### 2.2 The influence of extraction and refining process on the contents of PAH4

The extraction method of oils significantly influences the generation and contents of PAH4. Research indicates that sesame oil extracted by pressing method contains lower levels of PAH4 compared to that extracted by screw pressing, as the latter involves high temperatures leading to thermal oxidation and PAH4 formation (Lee et al., 2020). Ji et al. (2020) conducted a comparative study on the impact of three different processing methods on the generation of PAH4 in peanut oil. The results indicated significant differences in the PAH4 contents in the oils based on the processing methods, with the order of PAH4 levels as follows: hot pressing > solvent extraction > cold pressing. Furthermore, the study found that peanut oil directly extracted from skin peanuts exhibited significantly lower levels of PAH4 compared to peanut oil obtained after removing the peanut skins before pressing (Ji et al., 2020). This phenomenon may be attributed to the presence of antioxidant phenolic compounds in peanut skins, which can effectively scavenge free radicals, thereby reducing the generation of PAH4 (Xu et al., 2023; Zhao et al., 2017). The lower PAH4 contents in oil-fried peanuts with skins compared to de-skin peanuts further confirms this phenomenon (Zhao et al., 2017). The PAH4 contents in cold-pressed sunflower seed oil are 0.6 ng/g. Microwave pretreatment of sunflower seed oil can enhance its flavor. However, with prolonged microwave exposure, the PAH4 contents in sunflower seed oil significantly increased. Particularly, after a processing time exceeding 6 min, the PAH4 contents surpassed the limit of 10 ng/g, indicating a potential health concern (Yin et al., 2022). Moreover, prolonging the extraction temperature and time can also result in increased PAH4 contents (Ji et al., 2019; Yin et al., 2022).

The refining process of oils plays a crucial role in reducing PAH4 contents. As shown in Table 2, refining involves steps such as neutralization, decolorization, deodorization, among others, which effectively remove PAH4 from oils. For instance, the PAH4 contents in deodorized sesame oil are significantly lower than that in unrefined cold-pressed sesame oil (Manahi et al., 2024). Previous studies have shown that the refining process can reduce PAH4 contents in oils by 70.19 %–85.40 % (Cai et al., 2022), with decolorization and deodorization steps showing significant efficacy in PAH4 removal. The use of activated carbon during refining is recommended as an effective method to further decrease PAH4 contents in oils (Camargo et al., 2012; Shi et al., 2017; Singh et al., 2023). Additionally, storage conditions also impact the PAH4 contents, with an increase in total PAH4 levels in oils observed with prolonged storage temperature and time (Gong et al., 2019).

**Table 2:** The influence of extraction method and refining process on the contents of PAH4 (μg/kg) in oils.

Oil	Extraction method	Refining process	PAH4 type and contents					Reference
			BaA	Chr	BbF	BaP	PAH4	
Peanut oil	Roasting at 160 °C for 30 min	–	–	–	–	1.27	19.27	Ji et al. (2022)
	Roasting at 260 °C for 60 min		–	–	–	∼4.4	∼48	
Sesame oil	Plate-pressing	–	1.44	1.40	0.35	0.11	3.30	Lee et al. (2020)
	Screw-expeller	–	1.55	1.33	0.39	0.14	3.41	
Red pepper seed oils	Screw-expeller	Crude	5.8	14.2	25.2	21.5	66.7	
		Neutralization	1.6	2.6	4.8	2.3	11.3	
		Filtration	1.4	3.2	4.5	3.8	12.9	
		Evaporation	1.1	3.1	5.4	3.1	12.7	
Peanut oil extracted with skinned peanut	Hot pressed		4.60	3.39	2.90	4.09	14.99	Ji et al. (2020)
	Cold pressed		0.85	0.43	0.65	0.47	2.41	
	Solvent extract		1.70	0.99	1.39	0.89	4.98	
Peanut oil extracted with de-skinned peanut	Hot pressed		16.20	18.40	14.48	15.92	65.00	
	Cold pressed		0.99	0.52	1.13	0.57	3.12	
	Solvent extract		4.87	5.11	5.27	4.33	19.58	
Sesame oil	Un-roasting		2.46	1.09	1.06	0.63	5.24	Ji et al. (2019)
	Roasting at 160 °C for 20 min		2.96	2.34	1.80	0.91	8.01	
	Roasting at 180 °C for 20 min		3.07	2.46	1.81	0.93	8.27	
	Roasting at 200 °C for 20 min		3.33	2.50	1.92	1.17	8.92	
	Roasting at 200 °C for 40 min		4.37	3.28	2.94	2.02	12.61	
	Roasting at 200 °C for 60 min		6.61	5.03	4.24	2.66	18.54	
Camellia oil	–	Crude oil	7.99	13.82	7.67	6.51	35.99	Cai et al. (2022)
		Neutralization	8.74	15.09	7.96	7.44	39.23	
		Water wash	9.02	16.13	8.85	7.32	41.32	
		Decolorization	1.08	2.44	0.83	0.86	5.21	
		Deodorization	2.12	4.01	1.03	1.12	8.28	
		Refined oil	2.32	4.12	1.12	1.22	8.78	
Peanut oil		Crude oil	38.80	37.20	29.20	24.00	129.2	Ma et al. (2017)
		Neutralization	24.11	22.60	23.10	3.58	73.39	
		Decolorization	0.54	0.87	0.27	0.06	1.74	
		Deodorization	22.19	22.09	16.15	14.21	74.64	
Soybean oil		Crude oil	3.07	3.54	0.72	0.73	8.06	Hua et al. (2016)
		Neutralization	1.42	3.36	0.80	0.32	5.90	
		Decolorization	1.00	1.20	0.76	0.61	3.57	
		Deodorization	0.81	0.86	0.29	0.59	2.55	

### 2.3 The impact of cooking methods on the formation of PAH4 in oils

Current research on the formation of PAH4 in oils predominantly focuses on the influence of various cooking methods. It is widely accepted that high-temperature cooking methods, such as frying, grilling, and baking, serve as the primary pathways for PAH4 generation (Min et al., 2018; Samarajeewa, 2023; Xu et al., 2023). The temperature and duration of oils exposure during frying are recognized as critical factors affecting PAH4 formation, particularly evident in deep frying processes characterized by substantial oils consumption and elevated temperatures, leading to a significant rise in PAH4 levels. PAH4 tend to increase quickly when temperatures exceed 200 °C, as the intense heat accelerates oils oxidation and decomposition, facilitating the formation of polycyclic structures (Siddique et al., 2021). In grilling, incomplete fuel combustion results in smoke containing abundant PAH4, which can interact with surface oils on food items to produce additional PAH4 (Sumer and Oz, 2023). Similarly, high-temperature baking promotes oils oxidation and PAH4 formation, with increased PAH4 content observed, especially in cases of oils overuse. Conversely, low-temperature cooking methods like steaming and boiling yield lower PAHs levels. Steaming and boiling, for instance, effectively suppresses PAH4 formation due to reduced temperatures and limited oil-heat source contact (Hee et al., 2024). Microwave heating, known for its rapid and concentrated energy transfer, can mitigate PAH4 formation to some extent; however, prolonged high-temperature exposure may still generate PAH4 (Siddique et al., 2021). Prolonged heating durations also contribute to PAH4 accumulation by enhancing oil-air interactions, thereby facilitating PAH4 production. The oxidative stability of oils correlates with PAH4 formation (Zhu et al., 2018), with varying fatty acid compositions in different oil types influencing PAH4 generation. Oils rich in saturated fatty acids, such as coconut oil, exhibit superior stability at high temperatures and lower PAH4 production. Conversely, oils high in unsaturated fatty acids, like rapeseed and sunflower oils, are prone to oxidation and degradation, resulting in elevated PAH4 levels (Ma et al., 2021). Furthermore, the pH of the cooking system impacts PAH4 formation, with higher pH levels favoring the formation of H-PAHs (BbF and BaP) (Wongmaneepratip and Vangnai, 2017).

The generation of PAH4 during oils cooking processes involves complex chemical reactions, including the oxidation of fatty acids, free radical reactions, and cyclization reactions. Under high-temperature conditions, fatty acids are prone to oxidation and decomposition, yielding peroxides, alcohols, aldehydes, and olefins as intermediate products. These products further undergo intramolecular cyclization and Diels-Alder reactions to form PAH4. Studies have indicated a positive correlation between the degree of fatty acid oxidation and the production of PAH4, suggesting that more severe fatty acid oxidation leads to higher PAH4 levels (Xu et al., 2024). Additionally, the unsaturation of fatty acids plays a crucial role in PAH4 formation, as double bonds in unsaturated fatty acids are more susceptible to free radical reactions, thereby promoting PAH4 generation (da Silva et al., 2018). Furthermore, the chain length and branching structure of fatty acids also impact PAH4 formation, with longer chain lengths and branching structures facilitating PAH4 generation at high temperatures (Oz, 2021; Pei et al., 2023; Xu et al., 2024).

## 3 The determination of PAH4 in oils

### 3.1 Gas chromatography-mass spectrometry (GC-MS)

As shown in Table 3, gas chromatography-mass spectrometry (GC-MS) is a widely used and mature technology for the detection of PAH4 in oils, combining the efficient separation capability of gas chromatography with the strong identification ability of mass spectrometry. Commonly employed methods for sample preparation include liquid-liquid extraction (LLE) and solid-phase extraction (SPE). For instance, Lee et al. (2020) utilized a solvent system comprising n-hexane, DMF, and water for LLE, with C18 as the adsorbent for SPE. Separation was achieved using a DB-5 column, effectively detecting BaA, Chr, BbF and BaP with detection limits ranging from 0.02 to 0.05 μg/kg and quantification limits from 0.08 to 0.18 μg/kg. This method exhibited higher sensitivity for PAH4 detection compared to the use of acetonitrile for LLE (Ji et al., 2022). The novel approach utilizing a polydimethylsiloxane/pyrazine-based hyper-crosslinked polymer-coated stir bar sorptive extraction in conjunction with GC-MS presents numerous benefits for monitoring PAH4 levels in edible oils. These advantages encompass heightened sensitivity and precision facilitated by a highly porous and durable sorptive coating, enhanced extraction efficiency attributed to meticulously optimized extraction parameters, streamlined sample preparation procedures reducing the risk of analyte loss, and enhanced method robustness characterized by excellent reproducibility and the reusability of stir bars. This innovative method contributes to a more dependable and sustainable analytical strategy for PAH detection in intricate oil matrices (Han et al., 2025). Furthermore, triple quadrupole mass spectrometry (GC-QqQ-MS) is also widely utilized for efficient and accurate detection of PAHs in complex samples (Lan and Wu, 2023; Sun and Wu, 2020; Teng et al., 2019). The advantages of GC-MS lie in its high separation efficiency, strong qualitative capability, and high detection sensitivity, enabling precise analysis of PAH4 in complex samples. However, it comes with high equipment costs, relatively complex operation, and demands high technical proficiency from operators. Additionally, the sample preparation process is laborious, prone to errors, and entails relatively high operating costs.

**Table 3:** Determination of PAH4 in oils by HPLC and GC-MS.

Methods	Pretreatment	Detection parameters	LOD/LOQ (μg/kg)					Reference
				BaA	Chr	BbF	BaP	
GC-MS	LLE: n-hexane, DMF: water (9:1, v/v)	Column: DB-5 column (30 m × 0.25 mm).	LOD	0.04	0.05	0.04	0.02	Lee et al. (2020)
	SPE: C18	MS conditions: electron ionization (EI) mode and selected ion monitoring (SIM) mode.	LOQ	0.14	0.18	0.14	0.08	
GC-MS	LLE: acetonitrile	Column: DB-5MS column (60 m × 0.25 mm, 0.25 μm).	LOD	0.06	0.10	0.11	0.10	Ji et al. (2022)
	SPE: C18	MS conditions: electron ionization (EI) mode and selected ion monitoring (SIM) mode.	LOQ	0.20	0.33	0.37	0.33	
GC-MS	stir bar sorptive extraction with PDMS/HCPPz-TPB coating	Column: HP-5MS capillary column (30 m × 0.25 mm × 0.25 μm).	LOD	0.05	0.04	0.06	0.07	Han et al. (2025)
		MS conditions: electron ionization (EI) mode and selected ion monitoring (SIM) mode.	LOQ	0.18	0.12	0.20	0.25	
GC-QqQ-MS	LLE: Water, acetonitrile: acetone mixture (3:2, *v*/*v*)	Column: DB-5MS capillary column (30 m × 0.25 mm × 0.25 μm).	LOD	0.07	0.06	0.09	0.11	Sun and Wu (2020)
	SPE: EMR-Lipid	MS conditions: electron ionization (EI) mode and multiple reaction monitoring (MRM) mode.	LOQ	0.23	0.20	0.30	0.36	
UHPLC-FLD	LLE: n-hexane, DMF:water (9:1, v/v)	Column: Zorbax Eclipse PAH (100 × 2.1 mm, 1.8 μm).	LOD	0.08	0.09	0.30	0.08	da Silva et al. (2018)
	Dilution DMF:water (1:2, v/v)SPE: C18	Mobile phase: Acetonitrile (A) and water (B).	LOQ	0.25	0.30	1.00	0.25	
HPLC-FLD	LLE: n-hexane, DMF:water (9:1, v/v)	Column: C18 (250 × 4.6 mm, 5 μm).	LOD	0.23	0.30	0.07	0.10	Molle et al. (2017)
	SPE: C18	Mobile phase: Acetonitrile (A) and water (B).	LOQ	0.3	0.3	0.3	0.3	
HPLC-DAD	MSPE: Fe_3_O_4_@COF (TpDA)	Column: Hypersil GLOD column (150 × 4.6 mm, 3 μm).	LOD	0.42	0.18	0.32	0.33	Shi et al. (2018)
		Mobile phase: 5 % acetonitrile (A) in water and acetonitrile (B).	LOQ	1.40	0.60	1.06	1.12	
CWSFS	DMSO	Δλ = 63 nm	LOD	0.026	0.013	0.068	0.014	Wei et al. (2024)
			LOQ	0.087	0.043	0.227	0.047	

### 3.2 High-performance liquid chromatography (HPLC)

High-performance liquid chromatography with fluorescence detection (HPLC-FLD) is another commonly used method for PAH4 detection, combining the efficient separation capability of liquid chromatography with the high sensitivity of fluorescence detection, suitable for detecting PAH4 in food oils. Molle et al. (2017) employed a solvent system of n-hexane, DMF, and water for liquid-liquid extraction, followed by separation using a C18 column and acetonitrile-water mobile phase for PAH4 analysis. The detection limits were found to be 0.23, 0.30, 0.07, and 0.10 μg/kg for BaA, Chr, BbF, and BaP, with quantification limits all at 0.3 μg/kg. Lower detection and quantification limits were achieved using UHPLC-FLD, except for BbF (da Silva et al., 2018). In addition to FLD detectors, diode array detectors (DAD) have been utilized for PAH4 determination, providing compound spectral information beneficial for qualitative and structural identification of PAH4 in complex samples. However, DAD exhibits lower detection sensitivity compared to FLD, particularly for the sensitive detection of low levels of PAH4 (Shi et al., 2018).

### 3.3 Fluorescence spectroscopy

Traditional detection methods such as HPLC and GC-MS, while reliable, involve complex sample preparation processes, high costs, and lengthy analysis times, making them unsuitable for rapid screening. PAH4 compounds exhibit strong fluorescence properties, and fluorescence spectroscopy, known for its high sensitivity, is well-suited for PAHs detection in edible oils. Orfanakis et al. (2023) innovatively developed a fluorescence - based detection method for BaP in extra - virgin olive oil (EVOO) without sample pretreatment or extraction. Using a Jobin-Yvon Horiba Fluoro Max-P spectrometer at excitation wavelengths of 365 nm and 385 nm, and an emission wavelength range of 400–450 nm, the method achieved rapid and sensitive detection. Data analysis via partial least squares regression ensured accurate quantification. This method offers several advantages, including simplicity, speed, high sensitivity, environmental friendliness, and suitability for on-site detection, thereby ensuring that oil complies with food safety standards.

Moreover, researchers have developed a method that combines Constant Wavelength Synchronous Fluorescence Spectroscopy (CWSFS) with Back Propagation Neural Network (BPNN) machine learning algorithms for rapid quantitative analysis of PAH4 in edible oils. This method, from sample preparation to completion of analysis, takes less than 20 min, with data analysis requiring only 3 min. It demonstrates excellent stability, with recovery rates exceeding 97 % in various edible oil samples and relative standard deviations below 3.09 %. Compared to HPLC, no significant differences were observed, highlighting its potential as a rapid and reliable alternative for PAH4 analysis in edible oils (Wei et al., 2024).

### 3.4 Others

Electrochemical sensors have been utilized for the detection of PAH4, based on the oxidation-reduction reactions of PAH4 on the electrode surface. Qualitative and quantitative analysis of PAH4 is achieved by measuring the electrochemical signals such as current and potential generated from these reactions. Furthermore, electrode modification using nanomaterials and molecularly imprinted polymers can enhance sensor performance. While electrochemical sensors are widely applied for PAH4 detection in water (Beloglazova et al., 2018; Chen et al., 2022; Muñoz et al., 2018), their application in detecting PAH4 in oil matrices remains unexplored due to the complexity of lipid substrates. However, electrochemical sensors offer high sensitivity and selectivity, capable of detecting low concentrations of PAHs with rapid response and simple operation, making them suitable for on-site rapid detection. Therefore, electrochemical sensors hold great potential for the detection of PAH4 in oil matrices in the future.

Surface-enhanced Raman scattering (SERS)-based optical sensors are utilized for the detection of PAH4 by exploiting the surface plasmon resonance generated by plasmonic nanostructures (such as gold and silver), significantly enhancing the intrinsic Raman signals of PAH4 for highly sensitive detection (Du et al., 2016; Wang et al., 2020). This method features simple sample pretreatment, making it suitable for detecting complex matrices with high sensitivity capable of detecting extremely low concentrations of PAH4 (Chen et al., 2022). Su et al. (2021) developed a detection method based on liquid-interface SERS technology, which does not require sample pretreatment. By utilizing a three-dimensional nanostructure formed by self-assembled gold nanoparticles (GNPs), rapid and direct detection of BaP in edible oils was achieved. The entire detection process only takes 3 min, with a detection limit as low as 0.1 μg/kg. This method is applicable to various edible oils and has been successfully applied for the detection of BaP in real samples and the identification of waste oil adulteration, providing a reliable and rapid technical means for the supervision of oil quality and safety.

## 4 The mitigation of PAH4 in oils

In order to mitigate the formation of PAH4 compounds, it is essential to implement effective intervention measures. Subsequently, the effects of mitigating PAH4 in oils will be explored through discussions on raw material selection, processing technology, oils cooking, and antioxidants.

### 4.1 Selection appropriate oils

Due to the migration of PAH4 from the environment to oilseeds or oils, it is essential to avoid selecting contaminated oilseeds or oils as raw materials (Wen et al., 2017). For example, oilseeds grown in farmlands near industrial pollution sources or busy traffic areas are more susceptible to PAH4 contamination (Sampaio et al., 2021).

During thermal processing of food, the production of PAH4 is closely linked to lipids, with fatty acids exhibiting a higher susceptibility to PAH4 formation compared to glycerides. The higher the esterification degree of glycerides, the enhanced thermal stability, leading to reduced PAH4 formation (Xu et al., 2024). Additionally, selecting saturated fatty acids with high saturation levels and low susceptibility to oxidation as the fat source in food processing can significantly reduce the formation of PAH4 compounds. Min et al. (2018) utilized freeze-dried beef as a meat model system to investigate the formation of PAH4. Various concentrations and types of lipid precursors were incorporated and subjected to specific heating conditions for analysis. The findings revealed a significant increase in PAH4 formation with the addition of lipid precursors. Among the different types, methyl linolenate exhibited the highest PAH4 content, whereas methyl stearate showed the lowest. Electron spin resonance results indicated a potential connection between the impact of lipid precursors on PAH4 formation and lipid oxidation as well as free radical reactions.

### 4.2 Optimization of processing technology

High temperature is a key factor leading to fatty acid oxidation and the generation of PAH4 compounds in oils. Studies have shown that reducing processing temperatures and shortening high-temperature treatment times can effectively decrease fatty acid oxidation reactions, thereby reducing the formation of PAH4 compounds.

The levels of PAH4 in oils are significantly influenced by different extraction and purification processes. Cold pressing is a common method for sesame oil extraction, however, the lack of effective purification steps during this process results in higher PAH4 content in cold-pressed sesame oil. In contrast, refined and deodorized oils exhibit significantly lower levels of PAH4 compounds (Manahi et al., 2024). It is important to note that solvent extraction, while increasing oils extraction efficiency, may also elevate the degree of oil oxidation. Therefore, in practical production, the selection of extraction and purification processes should be carefully considered to achieve optimal removal of PAH4 compounds. The refining process presents a complex impact on PAH4 levels. Some steps may increase PAH4 contents. For example, neutralization and water wash steps in the refining process could lead to an increase in PAH4 levels in the oils (Cai et al., 2022). However, further processing steps such as decolorization and deodorization can effectively reduce PAH4 levels. Through neutralization and water washing, the PAH4 content in oils could be reduced by 42.2 %–98.7 % (Cai et al., 2022; Hua et al., 2016; Ma et al., 2017). Reducing the deodorization temperature and shortening the deodorization time appropriately can decrease the formation of PAHs while ensuring the quality of the oils.

Compared to reducing PAH4 through the refining process, it is more significant to decrease the PAH4 content before oils production. For instance, by altering the drying method of olive pomace to microwave drying, the PAH4 contents in the produced olive pomace oil can be reduced by 75 % (Kiralan et al., 2017). Additionally, the material and cleanliness of processing equipment may also impact the generation of PAH4. If there are residues of PAH-containing substances on the surface of the equipment, they may migrate into the edible oils during processing.

Adsorbents play a crucial role in removing PAH4 compounds during the refining process. The adsorption efficiency of three adsorbents (Notit-8015 activated carbon, WY activated carbon, and regular activated carbon) on PAH4 compounds was compared. These adsorbents can physically or chemically adsorb PAH4 compounds from oils, thereby reducing the PAH4 contents. It was found that regular activated carbon exhibited more significant adsorption efficiency. In the processing of sesame oil, the use of activated carbon as an adsorbent can effectively eliminate PAH4 compounds, enhancing the purity and safety of the oils (Shi et al., 2017). Additionally, some novel adsorbent materials, such as magnetic carbon nitride nanosheets, have shown promising application potential. With high specific surface areas and abundant active sites, they can rapidly and efficiently adsorb PAH4, offering a new option for controlling PAH4 during the processing of edible oils (Zheng et al., 2016).

### 4.3 Selecting appropriate cooking methods

Utilizing indirect heating, reducing direct contact between fats and flames, and preventing oil droplets from falling onto the heat source can effectively reduce the formation of PAH4 compounds. For instance, the impact of direct grilling versus indirect grilling methods on PAH4 formation were analyzed, and found that fewer PAH4 compounds were generated under indirect grilling conditions. This suggests that in the process of indirect grilling, separating the heat source from the food reduces fat dripping and smoke contact, thereby lowering the formation of PAH4 compounds (da Silva et al., 2018). Different cooking methods significantly influence the contents of PAH4 in grilled meat. Samples of grilled meat cooked using electric and gas heating sources exhibit lower PAH4 levels, while samples cooked using charcoal and wood heating sources show higher PAH4 levels. Specifically, the order of PAH4 content under different heating sources is charcoal > wood > gas > electric. The PAH4 contents in grilled chicken samples is generally higher than that in grilled beef samples, which may be related to the fat content of chicken and beef. Studies indicate that reducing the contact between fat and the heat source can decrease the generation of PAH4 (Akkaya et al., 2024). Furthermore, low-temperature cooking methods like steaming and boiling yield lower PAHs levels, which effectively suppresses PAH4 formation due to reduced temperatures and limited oil-heat source contact (Hee et al., 2024).

### 4.4 Antioxidants

The addition of natural or synthetic antioxidants, such as vitamin E, catechins, tea polyphenols, and rosemary extracts, during food processing can inhibit fatty acid oxidation reactions, thereby reducing the formation of PAH4 compounds (Gong et al., 2018; Keramat et al., 2023; Min et al., 2018). Additionally, many spices such as hogweed, rosemary, coriander have been proven to have inhibitory effects on the formation of PAH4 due to their antioxidant properties (Gong et al., 2018; Gulcin et al., 2019; Yu et al., 2023). For example, the presence of hogweed flavoring during the roasting process of sunflower seeds leads to a decrease in the contents of PAH4 (Shavali-gilani et al., 2023). In fried foods, rosemary extract can significantly reduce the formation of PAH4. The antioxidant components in rosemary, such as rosmarinic acid, have the ability to suppress lipid oxidation, consequently decreasing the formation of PAH4 (Gong et al., 2018).

Particularly, catechin exhibits a dual effect on PAH4 formation. At catechin concentrations exceeding 0.02 %, it effectively suppresses PAH4 generation. Conversely, when catechin levels are below 0.02 %, it not only fails to inhibit PAH4 formation but also contributes to its increase. This phenomenon is attributed to catechin undergoing oxidative decomposition reactions during high-temperature oxidation, leading to the generation of free radicals. When catechin levels are insufficient (<0.02 %), the excess free radicals attack triglycerides, initiating fatty acid oxidation and promoting PAH4 intermediates formation, consequently elevating PAH4 contents. Moreover, catechin undergoes cleavage and polymerization reactions at elevated temperatures, forming aromatic ring compounds that further enhance PAH4 levels. Additionally, oxidation products of catechin may undergo intramolecular cyclization to generate cyclic compounds, ultimately forming benzene rings. Molecular growth through the hydrogen extraction and acetylene addition (HACA) mechanism results in PAH4 formation, contributing to increased PAH4 content (Pei et al., 2023).

In reducing the content of PAH4, antioxidants, cellulose, and chitosan edible coatings play crucial roles, despite their different mechanisms of action. Antioxidants primarily inhibit the formation of PAH4 by scavenging free radicals, while cellulose and chitosan edible coatings reduce the permeation and formation of PAH4 through physical barrier and adsorption effects. Cellulose and chitosan edible coatings exhibit significant effectiveness in inhibiting PAH4. These coatings form a protective film that reduces oil permeation during the frying process, thereby lowering the likelihood of PAH4 entering the food with the oil. Additionally, the coatings can adsorb PAH4, especially high molecular weight PAH4, which are more toxic and carcinogenic. Furthermore, the coatings can serve as carriers for antioxidants, further enhancing the inhibition of PAH4 formation. Studies have shown that the use of cellulose and chitosan coatings in fried foods can significantly reduce the content of PAH4, thereby enhancing the safety of the food (Wang et al., 2023).

## 5 Summary and future prospective

**Fig. 2:**
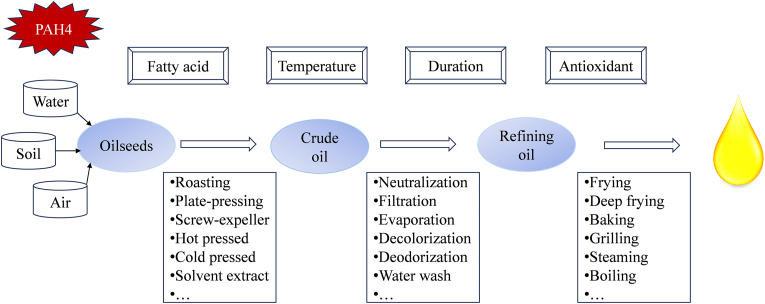
Generation of PAH4 from oilseeds to oil.

This review systematically examines the generation, detection, and mitigation of PAH4 in edible oils, focusing on their formation mechanisms during the transformation from oilseeds to oils. As illustrated in Fig. 2, the generation of PAH4 from oilseeds to oils is influenced by environmental factors, extraction and refining processes, as well as cooking methods. No matter each phase, the main influencing factors include fatty acid composition, temperature, duration, and antioxidants. The review also summarizes current detection techniques, including GC-MS and HPLC, and emphasizes the effectiveness of strategies such as selecting appropriate oils, optimizing processing techniques and using antioxidants in reducing PAH4 formation.

Despite its comprehensive coverage, the review notes a lack of in-depth exploration on PAH4 formation mechanisms in different oilseed crops and the generation mechanisms during the conversion process from oilseeds to oils. Additionally, existing detection technologies have certain limitations in practical applications, and the implementation of mitigation strategies may face challenges related to cost and technical feasibility in actual production.

Future research should focus on refining detection technologies to enhance the sensitivity and accuracy of PAH4 detection in edible oils and developing more efficient PAH4 removal techniques. There is also a need for a deeper understanding of PAH4 metabolism and toxicity in the human body to inform risk assessments and safety standards. The review stresses the importance of strengthening regulatory oversight across the entire production chain of edible oils to ensure consumer safety.

## Author contributions

Jiao-jiao Yin: Writing - Original Draft, Writing-review and editing, Conceptualization, Funding acquisition; Xiao-ming Jiang, Yi Nie and Wu Zhong: Formal analysis, Investigation; Xing-he Zhang, Pan Gao and Dong-ping He: Conceptualization.

## Funding

This study is supported by the Open Research Fund of the Key Laboratory of Edible Oil Quality and Safety, State Administration for Market Regulation (SYYKF202405).

## Declaration of competing interest

The authors declare that they have no known competing financial interests or personal relationships that could have appeared to influence the work reported in this paper.

## Data Availability

Data will be made available on request.
